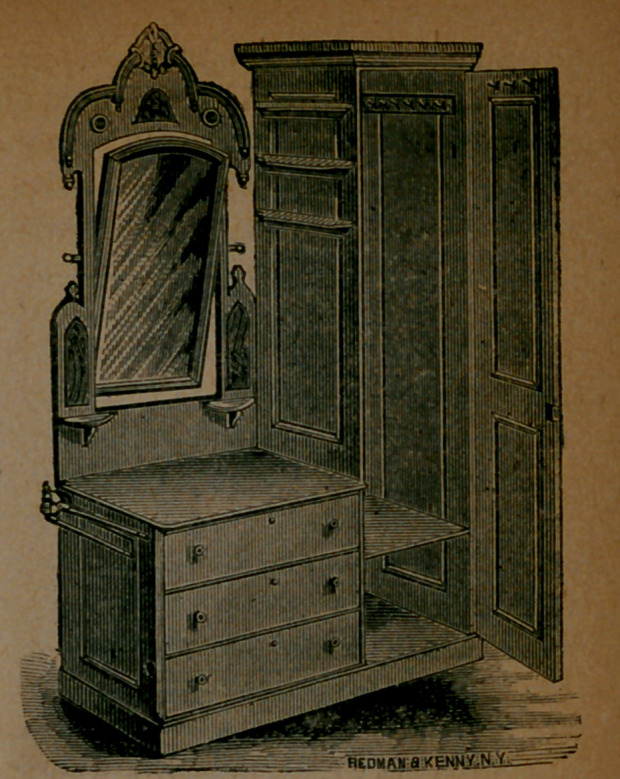# Decaying Teeth

**Published:** 1875-03

**Authors:** 


### DECAYING TEETH.

We gave last month our ideas in brief, as to the value of the dentifrice known as Dr. Lyon's Tooth Tablets. Since that issue we have conversed with a good many persons who have used this article, and have been pleased to find that our views are corroborated by the most competent judges. Several physicians and dentists assure us that they have known frail and delicate teeth to be saved from decay and injury by the use of these tablets.

Others have spoken of the agreeable odor imparted to the breath by their employment, as well as the cleanly and attractive appearance following their use. In fact, the testimony to their great value is well-nigh overwhelming; and we are fully convinced that no other article now in general use among us has like claims upon our regard, or is as largely recommended by those whose judgment is trustworthy in the matter.

Indeed, we should deem it remarkable if there were any difference of opinion concerning this excellent preparation. We know precisely of what pure and safe materials it is compounded, and with what infinite pains the good Dr. Lyon selects and prepares these choice substances.

We are not overstating the matter when we assert, that, if every man, woman, and child, in the land, were to use one of these tablets each night before sleeping, aided by a good stiff brush, decaying teeth would soon be almost unknown. How many of our readers—if any there be, who are negligent in this matter—will provide themselves and those under their care with this valuable preparation, and adhere earnestly to its use?

"The Economic."—We described the above useful and attractive article of furniture in our February number. The price is from $18 to $35. The manufacturer is A. E. Barnes, No. 438 Pearl Street, New York; and the trade supplies it all over the country. It is, as will be seen, a combined bureau, washstand, wardrobe, towel-rack, glass, and book-shelves; and is generally admired.

---

Asphalt is a most durable and impenetrable substance. Common brown paper, dipped in melted asphalt, is the most perfect substance to use as wrappers for silks or woolens which it is desirable to preserve from air and moisture and moths. A package may thus be made so secure that it may be soaked in water and its contents kept dry. Tubes may be made of paper thus saturated, which will last a long time as pipes for the conveyance of water. Anybody can make such tubing, and its lightness and cheapness will commend it for many uses.

## Figures and Tables

**Figure f1:**